# Supplementary material for: The Co-Design/Co-Development and Evaluation of an Online Frailty Check Application for Older Adults: Participatory Action Research with Older Adults
Source: Int J Environ Res Public Health. 2023 Jun 10;20(12):6101. doi: 10.3390/ijerph20126101 (PMC10297892; doi:10.3390/ijerph20126101)
Supplement: Supplementary file 1 [file ijerph-20-06101-s001.zip › ijerph-2418286-supplementary.pdf]

Online Supplementary Data

The Co-Design/Co-Development and Evaluation of an Online Frailty Check Application for Older Adults: Participatory Action Research with Older Adults

Bo-Kyung Son [1,2,3,*], Takahiro Miura [1,4], Ken-ichiro Yabu [1,5], Yuka Sumikawa [6], Dongyool Kim [7], Weida Lyu [1,3], Yingxue Yang [8], Moeko Tanaka [6], Tomoki Tanaka [1], Yasuyo Yoshizawa [1] and Katsuya Iijima [1,2,3]

[1] Institute of Gerontology, The University of Tokyo, Tokyo 113-8656, Japan; miu@iog.u-tokyo.ac.jp (T.M.); yabu@human.iog.u-tokyo.ac.jp (K.-i.Y.); weidalvleo@gmail.com (W.L.); tmk-tanaka@iog.u-tokyo.ac.jp (T.T.); yoshizawa@iog.u-tokyo.ac.jp (Y.Y.); iijima@iog.u-tokyo.ac.jp (K.I.)
[2] Institute for Future Initiatives, The University of Tokyo, Tokyo 113-0033, Japan
[3] Department of Geriatric Medicine, Graduate School of Medicine, The University of Tokyo, Tokyo 113-8655, Japan
[4] Human Augmentation Research Center (HARC), National Institute of Advanced Industrial Science and Technology (AIST), Kashiwa 277-0882, Japan
[5] Research Center for Advanced Science and Technology (RCAST), The University of Tokyo, Tokyo 153-8904, Japan
[6] Division of Health Sciences and Nursing, Graduate School of Medicine, The University of Tokyo, Tokyo 113-0033, Japan; sumi-tky@g.ecc.u-tokyo.ac.jp (Y.S.); sagefemme.dansante.m@gmail.com (M.T.)
[7] Department of Agribusiness Management, Faculty of International Agriculture and Food Studies, Tokyo University of Agriculture, Tokyo 156-8502, Japan; dongyool.kim30@gmail.com
[8] Graduate School of Education, The University of Tokyo, Tokyo 113-0033, Japan; yuki123yang@hotmail.com
* Correspondence: son@iog.u-tokyo.ac.jp; Tel.: +81-3-5800-6534

Supplementary Table S1. Items of 11 checks

11checks	Items		
Nutrition	**1. Awareness of healthy diet:** “Do you try to keep a healthier diet than other people of your age and gender?”	Yes	No
	**2. Balanced diet:** “Do you eat vegetables and either meat or fish on a daily basis?”	Yes	No
Oral function	**3. Chewing function:** “Can you chew hard foods like squid jerky or pickled radish?”	Yes	No
	**4. Swallowing function:** “Have you ever choked on tea or soup?”	No	Yes
Exercise habits and physical activities	**5. Exercise habit:** “Is there any exercise that you take for at least 30 minutes a day, twice a week or more, that you have engaged in for at least one year?”	Yes	No
	**6. Walking/physical activity:** “Do you walk (or engage in a similar activity) for at least one hour each day?”	Yes	No
	**7. Gait speed:** “Do you think you walk faster than other people of your age and gender?	Yes	No
Social/mental/ cognitive (subjective) factors	**8. Frequency of going out:** “Do you go out less often than you did last year?”	No	Yes
	**9. Eating together:** “Do you eat with others at least once a day?”	Yes	No
	**10. Awareness of vitality:** “Do you consider yourself vibrant?”	Yes	No
	**11. Awareness of forgetfulness:** “Do you worry more about forgetting things than anything else?”	No	Yes

Supplementary Table S2. Questionnaires of Reliability and interaction quality of online frailty checkup application

	Items (7 points)	FCSs n=23	Participants n=20	p
	**Online-onsite Reliability (points/ 21 points)**	15.0 (7.0-21.0)	16.0 (12.0-21.0)	0.058
Q1	I think the FC check provided over the online FC application is the same as onsite FC check program	4.0 (1.0-7.0)	6.0 (1.0-7.0)	0.076
Q2	Whenever I made a mistake using the online FC application, I could recover easily and quickly	6.0 (2.0-7.0)	6.5 (1.0-7.0)	0.551
Q3	This online FC application gave error messages by FCSs that clearly told me how to fix problems	6.0 (1.0-7.0)	7.0 (4.0-7.0)	p<0.001
	**Interaction Quality (points/ 28 points)**	24.0 (9.0-28.0)	27.0 (16.0-28.0)	0.040
Q4	I could easily talk to the FCSs (or participants) using the online FC application	6.0 (1.0-7.0)	7.0 (3.0-7.0)	0.166
Q5	I could hear the FCSs (or participants) clearly using the online FC application	6.0 (2.0-7.0)	7.0 (4.0-7.0)	0.050
Q6	I felt I was able to express myself effectively	6.0 (3.0-7.0)	7.0 (1.0-7.0)	0.042
Q7	Using online FC application, I can see the FCSs (or participants) as well as if we met in person	6.0 (3.0-7.0)	7.0 (3.0.-7.0)	0.010

Values are presented as median (min-max). Mann-Whitney test. Cronbach’s a = 0.809
